# Deregulation of Transcription Factor Networks Driving Cell Plasticity and Metastasis in Pancreatic Cancer

**DOI:** 10.3389/fcell.2021.753456

**Published:** 2021-11-23

**Authors:** Ruthger van Roey, Thomas Brabletz, Marc P. Stemmler, Isabell Armstark

**Affiliations:** Department of Experimental Medicine 1, Nikolaus-Fiebiger Center for Molecular Medicine, Friedrich-Alexander University of Erlangen-Nürnberg, Erlangen, Germany

**Keywords:** ADM—acinar to ductal metaplasia, PanIN—pancreatic intraepithelial neoplasia, PDAC—pancreatic ductal adenocarcinoma, transcription factors (TFs), cellular plasticity, epigenetics (chromatin remodelling), development

## Abstract

Pancreatic cancer is a very aggressive disease with 5-year survival rates of less than 10%. The constantly increasing incidence and stagnant patient outcomes despite changes in treatment regimens emphasize the requirement of a better understanding of the disease mechanisms. Challenges in treating pancreatic cancer include diagnosis at already progressed disease states due to the lack of early detection methods, rapid acquisition of therapy resistance, and high metastatic competence. Pancreatic ductal adenocarcinoma, the most prevalent type of pancreatic cancer, frequently shows dominant-active mutations in *KRAS* and *TP53* as well as inactivation of genes involved in differentiation and cell-cycle regulation (*e.g. SMAD4* and *CDKN2A*). Besides somatic mutations, deregulated transcription factor activities strongly contribute to disease progression. Specifically, transcriptional regulatory networks essential for proper lineage specification and differentiation during pancreas development are reactivated or become deregulated in the context of cancer and exacerbate progression towards an aggressive phenotype. This review summarizes the recent literature on transcription factor networks and epigenetic gene regulation that play a crucial role during tumorigenesis.

## 1 Introduction

Patients suffering from pancreatic cancer (PaCa) have the lowest overall survival rate compared to other cancer types in Europe, with roughly 7% surviving over 5-years (European Comission, 2020). Although rated as the ninth most common cancer in Europe, it is currently the fourth most common cause of cancer-related deaths and expected to rank even higher by 2025 (Ferlay et al., 2016). Despite the emergence of new treatment regimens, average survival rates only marginally increased in the past decades. The most prevalent form of PaCa is pancreatic ductal adenocarcinoma (PDAC), accounting for 90% of all diagnosed cases. Different PDAC precursor lesions have been identified with pancreatic intraepithelial neoplasia (PanINs) accounting for the major lesions which continuously progress through distinct stages (Hruban et al., 2007; Macgregor-Das and Iacobuzio-Donahue, 2013). Lineage tracing in mice revealed that acinar cells undergoing acinar-to-ductal metaplasia (ADM) have the greatest propensity to form PanINs, whereas an ADM-PanIN-PDAC route in human PaCa is still controversial (Kopp et al., 2012; Storz, 2017). Mutational events driving PDAC formation have been identified, such as genetic alterations in the proto-oncogenic *KRAS* in early PanIN lesions, inactivation of the tumor suppressor gene *CDKN2A* in intermediate/late lesions, and mutations in *TP53* and *SMAD4* during the transition to carcinoma (Goggins et al., 2000; Wilentz et al., 2000; Lüttges et al., 2001). Unfortunately, none of these genetic mutations have yet been proven targetable.

The main problem of PDAC is its early propensity towards metastasis together with the lack of early-stage diagnosis and limited treatment options due to rapid acquisition of therapy resistance. Besides the described genetic alterations, early malignancy and resistance are dependent on dysregulated epigenetic and transcriptional networks. These deregulations promote cellular plasticity, which helps tumor cells to adapt to novel environmental challenges during the metastatic cascade, to evade intrinsic control mechanisms, and dampen therapeutic efficacy (Orth et al., 2019). For a better prediction of disease progression and stratification of patient treatments, transcriptional profiling of resected PDAC tumors led to the identification of different molecular PDAC subtypes. Of those, two major subtypes with high tumor cellularity were described: pancreatic progenitor/classical and squamous/quasi-mesenchymal/basal-like (Collisson et al., 2011; Moffitt et al., 2015; Bailey et al., 2016; Puleo et al., 2018). Among these subtypes, the squamous type confers the most dismal prognosis and is associated with loss of endodermal cell fate (Bailey et al., 2016). In addition, this subtype is poorly-differentiated and highly chemoresistant (Chan-Seng-Yue et al., 2020). In contrast, the pancreatic progenitor subtype shows enrichment for the corresponding endodermal markers with a slightly better prognosis and is well-to-moderately differentiated (Bailey et al., 2016). Different samples from the same patient indicated that the pancreatic progenitor and squamous subtype can co-exist within the same tumor (Hayashi et al., 2021). Moreover, these subtypes are highly plastic and can interconvert, making it even more challenging to identify specific markers and subtype-specific treatment regimens (Lomberk et al., 2018; Brunton et al., 2020)

Transcription factors (TFs) are important actors in the spatio-temporal regulation of gene expression by directly binding *cis*-regulatory genomic elements (promoters and enhancers), recruiting cofactors (activators or repressors), and the core transcriptional machinery (Lee and Young, 2013). Together with other gene regulatory mechanisms, they drive cellular gene expression to orchestrate vital biological processes such as development, differentiation, cell cycle progression, tissue homeostasis, and cellular identity in a complex and tightly controlled manner. Deregulation of the delicate TF networks is a major cause of cancer and many other human diseases (Furney et al., 2006; Lee and Young, 2013). Specifically, TFs play a central role in all six hallmarks of cancer, *i.e.* sustained angiogenesis, endless replication, resisting cell death, insensitivity to anti-growth signals, self-sufficiency in growth signals, and activating invasion and metastasis (Hanahan and Weinberg, 2000, 2011). Of note, a staggering 20% of oncogenes encode TFs and TFs are terminal effectors in oncogenic signaling, thus representing important mediators in cancer (Lambert et al., 2018).

Several TFs orchestrating pancreatic organogenesis and driving pancreatic cell identity are deregulated in PDAC, strongly contributing to disease onset and progression. In the current review, we present an overview of our current understanding of transcriptional regulatory networks crucial in pancreas development, tissue homeostasis, and focus on recent findings illustrating how dysregulation of transcriptional networks promotes PDAC pathogenesis. In addition, we discuss the status of therapeutic strategies to target deregulated transcriptional networks and promising perspectives for the future.

## 2 Transcription Factors That Orchestrate Pancreas Organogenesis

The pancreas in the adult is comprised of an exocrine and endocrine compartment. Acini make up 90% of the cells in the mature organ and secrete nutrient-digestive zymogens, that are collected by a branched network of intralobular ducts for the release into the duodenum (Larsen and Grapin-Botton, 2017; Atkinson et al., 2020; Lorberbaum et al., 2020). The endocrine cells comprise 1–2% of the organ, are organized in islets of Langerhans and synthesize peptide hormones. They are essential for regulating blood glucose levels, produced by α- and β-cells, the main endocrine cell types that produce Glucagon and Insulin, respectively (Pan and Wright, 2011; Bastidas-Ponce et al., 2017; Larsen and Grapin-Botton, 2017). Pancreas organogenesis in mice starts at embryonic day (E)8.5 when the pancreas anlage is emerging as two independently forming dorsal and ventral buds that later fuse. This process is identified by *Pdx1* expression, which induces another key TF for pancreas formation, Ptf1α (p48) (Burlison et al., 2008; Shih et al., 2013). Two phases of pancreas organogenesis can be distinguished, starting with a primary transition (E8.5-E12.5) to specify pancreatic cell types and a secondary transition (E12.5-E17.5) to establish spatial organization of the tissue and cell maturation for generating numerous endocrine and exocrine cells (Bastidas-Ponce et al., 2017; Larsen and Grapin-Botton, 2017; Dumasia and Pethe, 2020). Initiation and maturation depend on an orchestrated network of TF activities.

**FIGURE 1:**
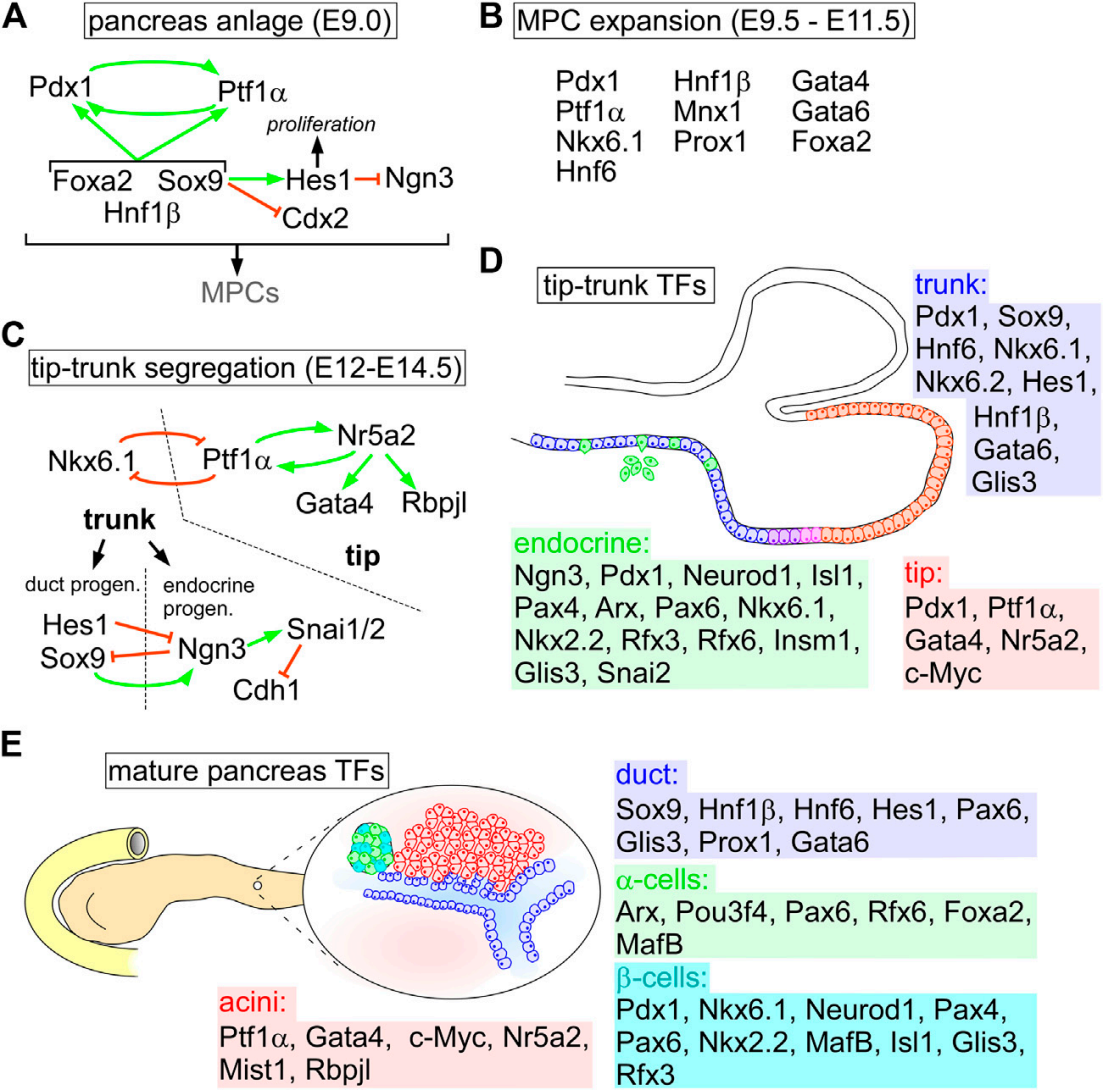
Transcription factor networks orchestrating pancreas specification and homeostasis. **(A)** Early events to form the pancreas anlage and MPC specification. **(B)** TFs involved in expansion of the MPC pool during primary transition. **(C)** TF networks to define tip and bipotent trunk domains. In the trunk additional networks are established for endocrine and duct specification. **(D)** Sketch of tip-trunk cell distribution in the pancreas progenitors highlighting TFs active in each domain’s networks. **(E)** overview on TFs of each cell compartment in the adult pancreas during homeostasis.

Analyses of gene expression patterns by *in situ* hybridization and immunofluorescence labeling revealed that the pancreas is specified by combined activities of Activin, Fgf2, retinoic acid, Bmp, Shh, and Notch pathways. The morphogenetic events involve the underlying mesoderm, endothelium and notochord (Deutsch et al., 2001; Chung et al., 2008; Pan and Wright, 2011; Shih et al., 2013; Xuan and Sussel, 2016; Lorberbaum et al., 2020). Pancreas identity is specified by increasing Pdx1 levels established by a feedback loop induced by Ptf1α (Ahlgren et al., 1996; Wiebe et al., 2007). Expression maintenance of these genes is controlled by a network orchestrated by Sox9, Hnf1β and Foxa2 (Shih et al., 2013; Bastidas-Ponce et al., 2017). Moreover, Sox9 is important to reinforce pancreatic identity by blocking *Cdx2* expression combined with activation of the Notch target *Hes1,* which in turn supports progenitor cell proliferation and repression of the endocrine cell inducer Ngn3 (Figure 1A) (Jensen et al., 2000; Ahnfelt-Rønne et al., 2012; Shih et al., 2015). This network generates a pool of multipotent progenitor cells (MPCs) that expand by combined activation of genes encoding Nkx6.1, Mnx1, Hnf1β, Hnf6 (Onecut1), Prox1, Foxa2, and Gata4/6 (Figure 1B) (Gittes, 2009; Pan and Wright, 2011). Many of these TFs have a pivotal role in early pancreas specification, as their loss results in organ agenesis or severe hypoplasia, including Ptf1α, Sox9, Mnx1, Gata4/6, Hnf1β, and Hes1 (Jonsson et al., 1994; Offield et al., 1996; Harrison et al., 1999; Li et al., 1999; Jensen et al., 2000; Haumaitre et al., 2005; Seymour et al., 2007, 2012; Watt et al., 2007; Shaw-Smith et al., 2014; Xuan and Sussel, 2016).

Once pancreas identity is established, branching morphogenesis in MPCs leads to separation into tip and trunk cells, precursors of acinar and ductal structures, respectively. Initially co-expressed between E10.5 and E13.5, *Nkx6-1* becomes restricted to trunk and *Ptf1a* to tip cells. Tip cells initiate *Myc* (c-Myc) expression, whereas trunk cells are defined by *Hnf1b*, *Sox9*, *Hnf6*, and *Hes1* gene activities (Bastidas-Ponce et al., 2017; Larsen and Grapin-Botton, 2017). Furthermore, expansion and maintenance of the exocrine compartment is further supported by inhibition of the Hippo pathway to repress endocrine specific TF genes, including *Pax6*, *Ngn3*, *Isl1,* and *Nkx6-1,* as well as *Gcg* and *Ins1/2* (Gao et al., 2013; Dumasia and Pethe, 2020). Consequently, active Hippo signals antagonize Yap activity promoting an endocrine fate (Rosado-Olivieri et al., 2019). In tip cells, Ptf1α induces *Nr5a2*, crucial for acinar identity, as Nr5a2 directly regulates *Ptf1a* in a feedback loop as well as *Gata4* and *Rbpjl* (Figures 1C,D) (Hale et al., 2014; von Figura et al., 2014). In addition to future acinar and duct cell fates, the endocrine compartment emerges in a few individual cells within the trunk that activate *Ngn3*, presumably by lateral inhibition orchestrated by the Notch pathway, as shown by lineage tracing in mice (Gu et al., 2002; Murtaugh et al., 2003; Magenheim et al., 2011). Ngn3^+^ cells delaminate from the trunk epithelium, subsequently cluster and form islets of Langerhans in the proximity of the tubular epithelium (Figure 1D) (Johansson et al., 2007; Pan and Wright, 2011; Shih et al., 2013). This process is reminiscent of epithelial-mesenchymal transition (EMT), by which epithelial cells lose the epithelial identity and apical-basal polarity to gain cell motility (for more details, see Box 1) (Johansson et al., 2007; Pan and Wright, 2011; Shih et al., 2013; Bastidas-Ponce et al., 2017). It involves coordinated expression of *Snai1* (Snail) and *Snai2* (Slug), EMT-TFs that are directly activated by Ngn3 and repress *Cdh1* (E-cadherin) (Figures 1C,D) (Rukstalis and Habener, 2007; Gouzi et al., 2011). Interestingly, another EMT-TF, Zeb1, is also expressed at low levels in the epithelial compartment of the developing pancreas. In contrast to the role of Snail and Slug during endocrine cell delamination, Zeb1 is crucial for proper lineage specification in correct ratios and for tissue homeostasis in the adult pancreas (Lasierra Losada et al., 2021). Temporal waves of TF expression initiate maturation of endocrine cells to ensure unidirectional unique cell type specification, including Neurod1, Insm1, and Rfx6, whose loss compromises islet cell identity and function (Bastidas-Ponce et al., 2017; Larsen and Grapin-Botton, 2017). Endocrine specification depends on repeated, transient rises in Ngn3 expression in the bipotent progenitor cells, that is regulated by *Pax6* activation, while maintained Pdx1 and Nkx6.1 levels are crucial for β-cell identity in the mature pancreas (Gannon et al., 2008; Schaffer et al., 2013) (Figure 1D).

BOX 1 **Epithelial-mesenchymal transition.**

EMT is an embryonic program that is essential for establishing the three germ-layers and other key morphogenetic events during development, but also becomes activated during wound healing. Besides its physiological function, EMT is hijacked during progression towards metastasis in various cancers (Nieto et al., 2016; Lu and Kang, 2019). The activation of EMT governs changes in cell fate, allowing (partial) transition of stationary epithelial cells towards a motile, invasive mesenchymal state (Johansson et al., 2007; Pan and Wright, 2011; Shih et al., 2013; Bastidas-Ponce et al., 2017). Recent findings show that the process of EMT is highly dynamic, representing a spectrum of intermediary states (Jolly et al., 2015; Nieto et al., 2016; Lambert et al., 2017; Aiello et al., 2018). Moreover, the reverse process mesenchymal-epithelial transition (MET) promotes metastatic colonization and outgrowth, highlighting the need for cellular plasticity during the metastatic cascade (Brabletz, 2012; Takano et al., 2016; Aiello et al., 2018). Various intrinsic and extrinsic signals can mediate the induction of EMT in cancer, often involving the activation of major signaling pathways, including TGFβ, HGF, BMP, PDGF, EGF, SHH, Notch, Integrin, WNT/β-catenin, and NF-κB (Taipale and Beachy, 2001; Heldin et al., 2012; Espinoza and Miele, 2013; McCormack and O’Dea, 2013; Gonzalez and Medici, 2014; Mihalko and Brown, 2018; Tam et al., 2020; Xu et al., 2020). Activation of EMT by any of these cascades often converges in the activation of a core set of EMT-TFs, including ZEB1/2, Snail (*SNAI1*), Slug (*SNAI2*), and Twist (Nieto et al., 2016; Stemmler et al., 2019). Consequently, EMT-TFs directly or indirectly downregulate genes that promote epithelial identity with apical-basal polarity, including *CDH1*, *EPCAM*, Claudins, and miR-200 family members (Brabletz and Brabletz, 2010; Dongre and Weinberg, 2019). Simultaneously, they activate mesenchymal genes that promote migration, invasion, and a front-rear polarity, including *CDH2*, *VIM*, *ACTA2* (α-SMA), *FN1*, and MMPs (Dongre and Weinberg, 2019).

In the adult pancreas, mature duct cells are maintained by continuous expression of trunk cell TFs, including Hnf6, Hnf1β, Sox9, Hes1, Pax6, Gata6, and Glis3, whereas mature acini express Ptf1α, Gata4, Mist1, and Nr5a2 (Shih et al., 2013; Bastidas-Ponce et al., 2017; Larsen and Grapin-Botton, 2017). Terminally differentiated β-cells are positive for Pdx1, Nkx6.1, Neurod1, Pax4/6, Rfx3, Nkx2.2, and MafA, whereas α-cells are defined by Arx, Pou3f4, Pax6, Rfx6, Foxa2, and MafB expression (Figure 1E) (Gittes, 2009; Pan and Wright, 2011; Shih et al., 2013; Cano et al., 2014; Dassaye et al., 2016; Bastidas-Ponce et al., 2017; Larsen and Grapin-Botton, 2017; Dumasia and Pethe, 2020; Jennings et al., 2020). Besides these regulatory circuits of TFs, correct pancreas progenitor formation, MPC identity, islet specification, and maintenance of individual cell types require epigenetic regulation and the activity of PcG proteins (Dumasia and Pethe, 2020). Deregulation of the established networks is an inevitable event in tumorigenesis and fosters disease progression.

## 3 Deregulated Expression of Transcription Factors in Tumorigenesis

### 3.1 Transcription Factors Driving Pancreatic Ductal Adenocarcinoma Initiation

PDAC is considered to emerge from a sequential progression of pre-neoplastic precursor lesions. Different histological types of putative precursor lesions have been described: PanIN, intraductal papillary mucinous neoplasia (IPMN), and pancreatic mucinous cystic neoplasm (MCN) (Hruban et al., 2000, 2007). PanIN lesions represent the most extensively studied precursors of PDAC and are categorized from PanIN1 to PanIN3, that accumulate progressive features reflecting increasing dysplastic morphology and acquisition of genetic alterations (Hruban et al., 2000, 2001; Maitra et al., 2003; Hezel et al., 2006; Guerra et al., 2007). Nevertheless, the cell of origin responsible for the initiation and early progression of PDAC remains undetermined. Despite the phenotypic similarity of these benign precursor lesions to ducts, mutant *Kras* expression in adult mouse ductal cells driven by *CK19* failed to induce PDAC, challenging the ductal origin of PDAC (Brembeck et al., 2003; Ray et al., 2011). Data from genetically engineered mouse models (GEMMs) suggest that the expression of oncogenic *Kras* in acinar cells induces transdifferentiation to duct-like cells during ADM. Although still debated, several lines of evidence suggest that this process precedes the formation of PanIN lesions and ultimately causes PDAC (Carriere et al., 2007; Guerra et al., 2007; Zhu et al., 2007; De La et al., 2008; Habbe et al., 2008; Morris et al., 2010; Kopp et al., 2012; Reichert et al., 2016). For example, analyses of patients with familial pancreatic cancer show that PanIN lesions, as well as ADM, and atypical flat lesions, can be found in human specimens (Brune et al., 2006; Zhu et al., 2007; Shi et al., 2009; Mazur et al., 2010; Aichler et al., 2012; Hidalgo-Sastre et al., 2016). Moreover, besides the classical PanIN-to-PDAC progression model, PDAC initiation was demonstrated to evolve separately from acinar or duct cells in a PanIN-independent mechanism (Ferreira et al., 2017). Likewise, expression of *Kras* ^*G12D*^ in combination with haploinsufficiency of *Smad4* leads to a sequential progression of MCN lesions towards a distinct class of PDAC (Izeradjene et al., 2007). Based on oncogenic mutations, TF networks become deregulated and cells start to transdifferentiate in multiple ways in favor of tumor progression.

Transdifferentiation or loss of cellular identity is a crucial feature at the onset of cancer formation (Slack, 2007; Stanger and Hebrok, 2013; Xiong et al., 2019). Upon injury or inflammation (pancreatitis) in mice, acinar cells can dedifferentiate towards a duct progenitor-like state, transiently expressing acinar, ductal, or early precursor markers to replenish the pancreas during tissue regeneration (Parsa et al., 1985; Song et al., 1999; Miyamoto et al., 2003; Jensen et al., 2005; Means et al., 2005; Kopp et al., 2012; Stanger and Hebrok, 2013; Storz, 2017). This involves the re-expression of progenitor and lineage-specific TFs and subsequent re-differentiation, demonstrating cellular plasticity induced upon injury. Acinar cell identity in mice is maintained by several cooperating TFs, such as Ptf1α and Mist1 (Pin et al., 2001; Rose et al., 2001; Pan and Wright, 2011; Martinelli et al., 2013; Bastidas-Ponce et al., 2017; Larsen and Grapin-Botton, 2017). Downregulation of these TFs results in the acquisition of progenitor cell characteristics and increased ADM and PanIN formation, highlighting the importance of maintained expression of these identity factors to prevent tumor initiation (Miyamoto et al., 2003; De La et al., 2008; Shi et al., 2009; Flandez et al., 2014; von Figura et al., 2014; Krah et al., 2015). In line with that, oncogenic *Kras* expression prevents the acinar re-differentiation and helps to maintain a ductal phenotype after acute inflammation, *e.g.* during pancreatitis. This suppressed re-differentiation promotes PanIN progression (Morris et al., 2010; Collins et al., 2012). Furthermore, during the ADM process in PDAC GEMMs, TFs involved in MPC specification or in ductal identity maintenance are (re-)expressed, including Pdx1, Hes1, and Sox9 (Song et al., 1999; Miyamoto et al., 2003; Jensen et al., 2005; Seymour et al., 2007; Kopp et al., 2012). Examples of the role of specific TFs that become deregulated during the early event of transdifferentiation and tumorigenesis are discussed in more detail.

#### 3.1.1 Gata6

Initially, Gata6 was presented as an important regulator of early pancreas specification and cell type differentiation, showing a partially overlapping expression with Gata4 (Ketola et al., 2004; Decker et al., 2006). Recently, Gata6 was demonstrated to be required for terminal differentiation and homeostasis of acinar cells and establishment of polarity (Martinelli et al., 2013). Evidently, *Gata6* inactivation induces massive loss of acinar cells and fosters ADM in the pancreas (Martinelli et al., 2013). In addition, *Gata6* ablation accelerates Kras^G12D^ driven tumorigenesis, demonstrating that Gata6 maintains acinar differentiation by driving expression of acinar master TFs and suppressing ectopic programs in the pancreas. Hence, in this context, Gata6 functions as a tumor suppressor (Martinelli et al., 2016). In fact, *GATA6*, among other genes encoding endodermal cell-fate determination TFs, is silenced via promoter hypermethylation in the squamous subtype of PDAC (Bailey et al., 2016; Seino et al., 2018). In line with that, *GATA6* expression was preferentially detected in well-differentiated low-grade tumors upon transcription profiling (Collisson et al., 2011; Moffitt et al., 2015; Diaferia et al., 2016). Interestingly, silencing of *Gata6* and the subsequent loss of acinar differentiation was observed during nicotine administration in mice, providing a possible link to cigarette smoking, which is a major risk factor contributing to pancreatitis and PDAC initiation (Hermann et al., 2014; Weissman et al., 2020). These findings altogether emphasize the importance of Gata6 maintenance to prevent tumor initiation and progression towards PDAC.

#### 3.1.2 Mist1

Mist1 is another acinar specification TF that is crucial for acinar cell maturation, function, stability, and identity and is involved in establishing granule organization and exocytosis pathways (Pin et al., 2001; Johnson et al., 2004; Direnzo et al., 2012). In the absence of Mist1 in pancreata with a Kras^G12D^ mutation, destabilization of the acinar phenotype leads to acceleration of PanIN formation (Shi et al., 2009). Furthermore, in cell culture models Mist1 was shown to reduce acinar cell proliferation rates by activating p21 (CIP1/WAF1) (Jia et al., 2008). Data from a 3D ADM culture model revealed that forced expression of *Mist1* attenuates Kras^G12D^-induced ADM and PanIN formation (Shi et al., 2013). Activation of *Mist1* upon orthotopic transplantation of murine PDAC cells rescues the acinar gene expression program (Jakubison et al., 2018). Overall, the maintenance of a differentiated acinar identity by Mist1 protects acinar cells from early tumorigenesis.

#### 3.1.3 Ptf1α

Ptf1α maintains acinar cell identity and restrains Kras-mediated tumorigenesis (Rose et al., 2001; Thompson et al., 2012; Krah et al., 2015; Hoang et al., 2016). Nevertheless, *Ptf1a* is downregulated during inflammation-induced ADM and in acinar cells transformed by Kras^G12D^ and Notch co-activation (Molero et al., 2007; De La et al., 2008). Specifically, downregulation of *Ptf1a* is a necessary and rate-limiting step in ADM and neoplastic progression to PanINs and PDAC to overcome the Ptf1α-mediated maintenance of acinar gene signatures and quiescence in mice (Krah et al., 2015). Additionally, *Ptf1a* was shown to be epigenetically silenced in murine ADM and PDAC cells harboring an oncogenic *Kras* allele (Benitz et al., 2016). Moreover, the sustained expression of *Ptf1a* prevents and reverts Kras-driven pancreas tumorigenesis, rescues the acinar gene program in PDAC cells, and can inhibit tumor growth (Jakubison et al., 2018; Krah et al., 2019). These examples highlight the role of Ptf1α as a key transcriptional regulator of acinar cell identity rendering differentiated acinar cells less sensitive for cancer initiation.

#### 3.1.4 Pdx1

In the adult pancreas, the primary function of Pdx1 is the specification and maintenance of mature β-cells (Ahlgren et al., 1998; Gannon et al., 2008; Gao et al., 2014). During tumor formation, *Pdx1* is upregulated in ADM and PanINs upon overexpression of TGFα or expression of oncogenic *Kras* (Song et al., 1999; Hingorani et al., 2003; Park et al., 2011). In addition, gain- and loss-of-function analyses in human PDAC cell lines resulted in increased proliferation and invasion potential in the presence of PDX1. In contrast, its loss decreases cell survival and tumor growth *in vivo*, suggesting that PDX1 acts as an oncogene (Liu et al., 2008). In line with that, persistent Pdx1 expression in the normal pancreas promotes ADM induction via Stat3 activation. Simultaneous depletion of Stat3 blocks ADM formation (Miyatsuka et al., 2006). Despite its oncogenic function, *Pdx1* often becomes downregulated by hypermethylation during progression towards the squamous and more aggressive subtype of PDAC. Conversely, PDX1 is part of a transcriptional network determining pancreatic endoderm cell fate and its presence results in a better prognosis in the pancreatic progenitor subtype (Bailey et al., 2016). In line with this conflicting data, Pdx1 was demonstrated to act as context-dependent TF during PDAC initiation and progression. Pdx1 switches from a safeguard of acinar cell identity during early tumorigenesis to an oncogene after the establishment of ADM (Roy et al., 2016). In summary, Pdx1 has two opposing functions that are activated in a context- and progression-dependent manner, emphasizing the necessity for more detailed analyses to better understand its bipartite function and prognostic value.

#### 3.1.5 Sox9

Expression of Sox9 in the adult pancreas is restricted to cytokeratin-positive duct cells including centroacinar cells (Miyamoto et al., 2003; Seymour et al., 2007; Furuyama et al., 2011; Shroff et al., 2014). During tumor formation, it was shown that *Sox9* is induced in ADM and PanINs and is maintained in the pancreatic progenitor PDAC subtype (Morris et al., 2010; Kopp et al., 2012; Prevot et al., 2012; Meng et al., 2014; Grimont et al., 2015). Importantly, ADM and PanINs originating from the acinar compartment require ectopic induction of *Sox9*. Specific depletion of Sox9 from acinar cells efficiently blocks Kras-mediated PanIN formation in mouse models (Kopp et al., 2012). Furthermore, co-expression of oncogenic *Kras* and wild-type *Sox9* promotes induction of precursor lesions from the acinar compartment (Kopp et al., 2012). Mechanistically, efficient repression of acinar genes and activation of ductal/progenitor genes in cells that undergo ADM is dependent on the combined expression of *Sox9* and *Hnf6*, as *Hnf6* overexpression also triggers ADM in mouse acinar cell lines and upon adenoviral gene delivery *in vivo* (Prevot et al., 2012). During pancreatitis, inflammation-induced EGFR signaling was shown to induce *Nfatc1* and *Nfatc4* expression, leading to ADM and PDAC progression due to upregulation of *Sox9* (Chen et al., 2015; Hessmann et al., 2016). In addition, SOX9 may play a role in the IPMN-PDAC route, however, conflicting evidence have been observed. Some studies identified a gradual decrease in SOX9-positive cells in IPMNs during progression, while others report constant or even elevated SOX9 expression in both low-grade and high-grade IPMNs compared to the normal pancreas (Tanaka et al., 2013; Shroff et al., 2014; Gnerlich et al., 2019). In mice, *Arid1a* deficiency in the Kras^G12D^ pancreas results in reduced Sox9 expression and less PanINs, but increased IPMN and PDAC formation. Simultaneous *SOX9* overexpression does not affect IPMN incidence, but reduces PDAC formation, demonstrating that Sox9 is a major downstream target of Arid1a and prevents tumor progression by promoting ductal differentiation (Kimura et al., 2018). Conclusively, Sox9 is a crucial mediator of ductal- or progenitor-like identity. Due to its embedding in multiple signaling pathways and feedback loops in cell-type specification, its deregulated expression is ultimately linked to early tumorigenesis.

#### 3.1.6 Hes1

In the adult pancreas, the expression of the Notch target *Hes1* is limited to centroacinar and ductal cells associated with progenitor cell function (Miyamoto et al., 2003; Kopinke et al., 2011). Upregulation of *Hes1* by active Notch signaling was observed during ADM and PanIN formation (Hingorani et al., 2003; Miyamoto et al., 2003; Jensen et al., 2005; De La et al., 2008; Plentz et al., 2009). Moreover, Notch-induced Hes1 was suggested to control the expansion of an undifferentiated precursor cell population, thereby promoting Kras-mediated tumor initiation and progression (Miyamoto et al., 2003; Jensen et al., 2005; De La et al., 2008). In fact, acinar-specific expression of mutant *Kras* induces *Hes1* expression along with ADM and PanIN formation. In this context, Notch activation was shown to sensitize acinar cells to mutant *Kras*-induced ADM/PanIN initiation and progression (De La et al., 2008; Nishikawa et al., 2019). Interestingly, Elastase-mediated Hes1 depletion blocks the progression from ADM to PanINs, combined with a re-differentiation to acinar cells (Nishikawa et al., 2019). However, the role of Hes1 is likely more complex as in another mouse model using Ptf1α-mediated *Hes1* ablation and oncogenic *Kras* induction, loss of *Hes1* displayed increased ADM formation and accelerated PDAC tumorigenesis. Reduced numbers of high-grade PanINs were detected in this model, hinting towards tumor formation from a direct ADM-to-PDAC route that skips precancerous PanIN lesions (Hidalgo-Sastre et al., 2016). These findings convey that context-specificity and maintained activity of Notch and Hes1 during homeostasis are essential regulators of tumor initiation.

**FIGURE 2:**
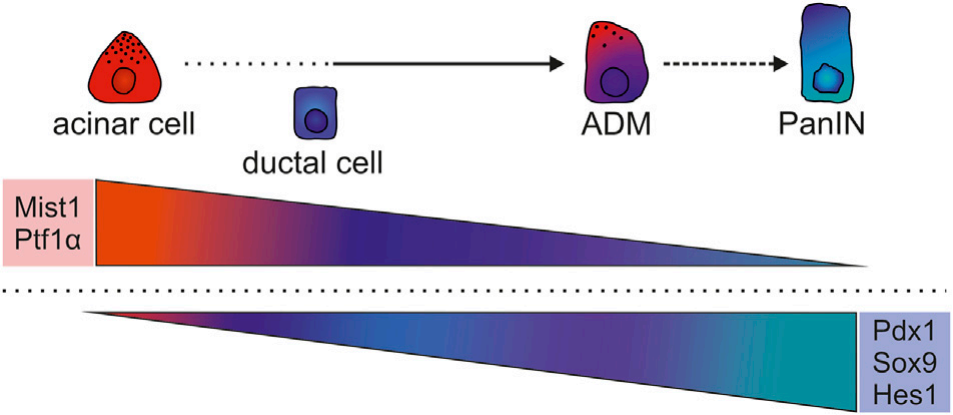
Modulated expression of key TFs of the process of transdifferentiation during ADM. Differentiated acinar cells are specified and maintained by Gata4, Ptf1α, Mist1, and others, whereas duct cells depend on Sox9, Hes1, and others. During ADM several duct-specific TF networks are induced including Sox9 and Hes1, while acinar-specific networks involving Ptf1α and Mist1 are collapsing. ADM cells also adopt non-duct like features by activation of Pdx1, gaining more progenitor-like characteristics. Although Gata6 is required for acinar specification, but absent in mature acinar cells, its continuous expression prevents ADM.

In summary, PDAC formation depends on early pre-neoplastic events like ADM, which relies on the downregulation of TFs that control acinar cell identity, including Gata6, Mist1, and Ptf1α, and a gain of TFs that promote duct or MPC-like specification, including Pdx1, Sox9, and Hes1 (Figure 2). However, some controversies and the incomplete understanding of the cellular origin of PDAC warrant further analyses to decipher the TF networks that are active during early tumorigenesis.

### 3.2 Transcription Factor Alterations Driving Pancreatic Ductal Adenocarcinoma Progression and Metastasis

Profiling of human PDAC specimens led to the identification of several PDAC subtypes (Collisson et al., 2011; Moffitt et al., 2015; Raphael et al., 2017; Puleo et al., 2018). The classification in the different studies largely overlap with one another (Collisson et al., 2019). Unsupervised clustering of PDAC tumors with high tumor cellularity identified the pancreatic progenitor and squamous subtype, suggesting that only these subtypes define the tumor compartment (Moffitt et al., 2015; Bailey et al., 2016; Puleo et al., 2018). Histopathologic evaluation revealed that tumors belonging to the pancreatic progenitor subtype are moderate-to-well differentiated, whereas the squamous subtype is poorly-differentiated (Puleo et al., 2018). Transcriptional network analysis of resected human PDAC specimens identified that the pancreatic progenitor subtype is enriched for TF transcripts pivotal for specifying pancreas cell-fate (*e.g. PDX1*, *HNF4A*, *HNF1B*, *HNF1A*, *FOXA2*, *FOXA3*, *HES1*, and *MNX1*) (Bailey et al., 2016). The squamous subtypes shows enriched gene networks involved in TGFβ signaling, *MYC* activation, inflammation, metabolic programming, and the upregulation of ∆Np63 and its targets. Multi-omics analyses of 24 patient-derived xenografts (PDXs) recapitulated the presence of the pancreatic progenitor and squamous subtype (Lomberk et al., 2018). Activated genes in the pancreatic progenitor subtype are mainly involved in pancreas development (*e.g. GATA6*, *BMP2*, *PDX1*, and *SHH*) and Ras signaling (*e.g. KITLG* and *RASA3*). The squamous subtype shows enrichment for pathways with strong oncogenic potential (*e.g.* PI3K-AKT, Hippo, and WNT), EMT (*e.g.* TGFβ signaling) (Box 1) and deregulation of genes involved in cell proliferation, differentiation and apoptosis (*e.g. YAP1*, *CD44*, *MYC,* and *E2F7*). These findings connect PDAC subtypes and differentiation states to deregulated signaling pathways. The deregulation of a subset of TFs play a pivotal role in facilitating a subtype switch to the more aggressive squamous phenotype by altering transcriptional regulatory networks. We will discuss these TFs and their effects upon deregulation.

#### 3.2.1 Subtype-specific Transcription Factors

The oncogenic *KRAS* mutation is found in over 90% of PDAC patients and results in the persistent stimulation of downstream signaling leading to sustained cell proliferation, transformation, migration, and survival (Biankin et al., 2012; Buscail et al., 2020). Although Ras signaling is enriched in the pancreatic progenitor subtype, the activation of certain KRAS downstream mediators is able to foster the transition towards the squamous subtype. Elevation of Etv1, a downstream target of Kras, promotes stromal expansion and metastases through S*parc* and *Has2* activation in tumors generated by orthotopic transplantation of KPC cells (Kar and Gutierrez-Hartmann, 2013; Heeg et al., 2016). *Etv1* overexpression induces all core EMT-TFs (Box 1) and molecular markers associated with the mesenchymal phenotype (*e.g. Vim*, *Mmp3*, and *Mmp9*), whereas knockdown of *Etv1* reduces Zeb1 levels. Using human PaCa cell lines *in vitro* it was shown that elevation of HAS2 is able to fuel a self-enforcing feedback loop of CD44 and ZEB1 that involves differential splicing of CD44 by ESRP1, further promoting EMT (Preca et al., 2015, 2017). In addition, EMT and enhanced invasion can be activated by increased *MAZ* expression in human PaCa cell lines*.* MAZ acts downstream of KRAS and facilitates CRAF-MAPK signaling involving PAK and suppression of AKT/PKB (Maity et al., 2018). Moreover, the upregulation of MAPK or inactivation of *TP53* leads to the overexpression of *KLF7*, promoting tumor growth and metastasis in mice (Gupta et al., 2020). Expression of KLF7 activates IFN-stimulated genes and stabilizes Golgi integrity and thus protein glycosylation to enhance the secretion of cancer-promoting growth factors. In cooperation with *Myc* Yap1 maintains the expression of metabolic genes required for proliferation and survival (Murakami et al., 2019). Ablation of *Yap1* in a PDAC mouse model leads to the downregulation of *Myc*, inducing growth arrest and apoptosis (Murakami et al., 2019). Interestingly, a subset of tumor cells was able to restore Myc levels allowing cell survival through the induction of genes encoding EMT-TFs Snail, Zeb2, Twist2, and the stemness factor Sox2, thus compensating for *Yap1* loss.

**FIGURE 3:**
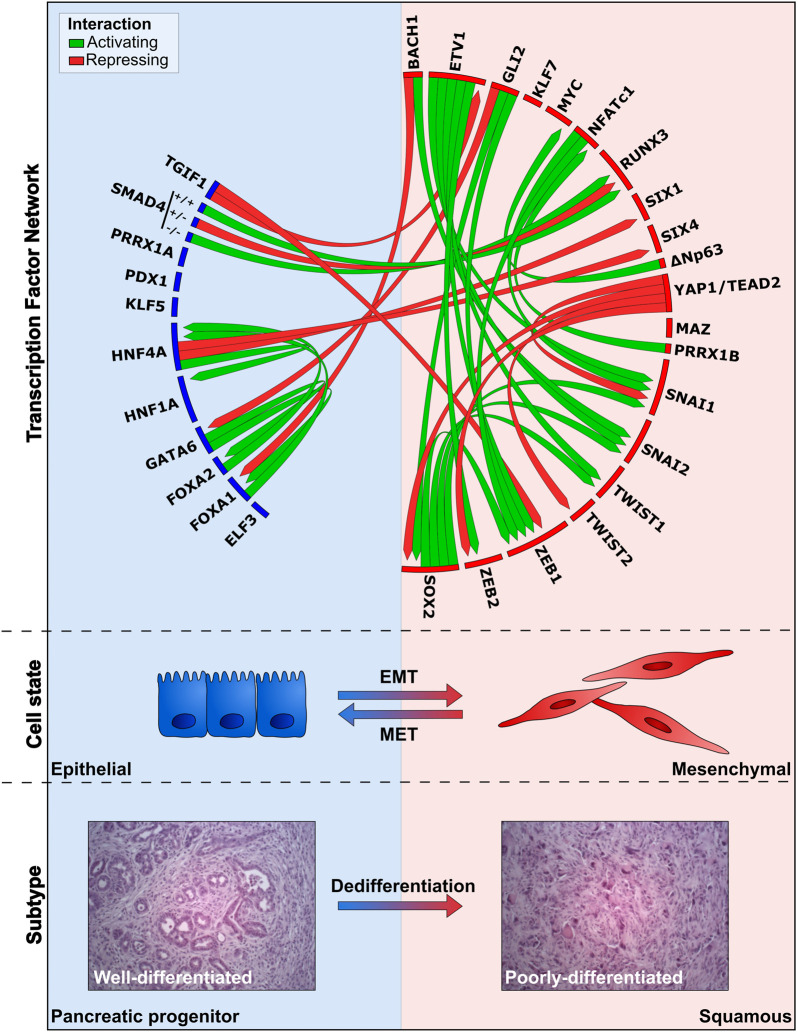
Overview of TF networks active during PaCa progression, EMT, and metastasis. The deregulation directly or indirectly affects the expression of other TFs, thereby promoting or inhibiting the differentiation/EMT state. TFs depicted in the blue box are associated with the epithelial cell-state/pancreatic progenitor subtype (E-PP), whereas the red box shows TFs linked to the mesenchymal-state/squamous subtype (M-S). Links resulting in activation and repression of TFs in the network are indicated by green and red lines, respectively. Expression of E-PP TFs in the blue box promote the differentiated endoderm/epithelial identity, block dedifferentiation, and prevent activation of stemness/EMT/dedifferentiation TFs. Activation of M-S TFs is associated with a mesenchymal identity, promotes dedifferentiation and activation of EMT and stemness. TFs without connections in the network have been associated with specific subtypes, but how they integrate into the network is poorly understood. H&E images depict well-differentiated and poorly-differentiated tumors, derived from KPC primary pancreatic tumors, reflecting E-PP and M-S phenotypes (adapted from Krebs et al. (2017)).

Multiple studies show that the pancreatic progenitor subtype is KRAS-dependent, whereas the squamous subtype is less dependent on KRAS (Singh et al., 2009; Collisson et al., 2011; Ischenko et al., 2021). Moreover, CRISPR/Cas9-mediated *Kras* knockout in tumor cells derived from the KPC mouse model showed pathway enrichment for EMT and TGFβ signaling, hinting that ablation of *Kras* drives a subtype switch towards the squamous subtype (Ischenko et al., 2021). Secondary ablation of *Kras* ^*G12D*^ in established tumors of a GEMM with doxycycline-inducible *Kras* ^*G12D*^ and conditional *Tp53* inactivation leads to complete regression (Kapoor et al., 2014). Although these initial results are promising, the majority of mice show relapse and exhibit poorly-differentiated pancreatic tumors. The survival of tumor cells in this model in the absence of *Kras* is mediated by the upregulation of the transcriptional coactivator Yap1, a downstream mediator of the Hippo signaling cascade, and Tead2, forming Yap1/Tead2 complexes coordinating downstream gene expression. Other compensatory mechanisms have been identified, including the induction of the transcriptional repressor *Gli2*, a downstream mediator of the Shh pathway, upon *in vitro* Kras^G12D^ ablation (Adams et al., 2019; Ischenko et al., 2021). *Gli2* induction rescued viability and induced upregulation of squamous-specific gene signatures (*e.g. Vim* and *Zeb1*). Moreover, *GLI2* induction in human PaCa cell lines promotes a gene signature switch from the pancreatic progenitor towards the squamous subtype, accompanied by a decrease in epithelial identity markers (E-cadherin, ESRP1, GATA6, and SHH) and enrichment in expression of EMT/stemness markers (ZEB1, VIM, CK14, SOX2, and CD44). Primary tumor growth and metastatic outgrowth can be suppressed by ablation of *SPP1*, a downstream target of GLI2, emphasizing its role in promoting tumor aggressiveness. These findings demonstrate that aberrant activation of several TFs exacerbate PDAC progression (Table 1) with various degrees of KRAS-dependency. Interestingly, these deregulations frequently mediate the indirect upregulation of EMT-TFs (ZEB1/2, Snail, Slug, and Twist) and stemness factors (SOX2 and CD44) (Figure 3, Box 1). These findings corroborate that the induction of the reversible EMT program promotes an aggressive PDAC phenotype by enabling cellular plasticity, metastasis formation, chemoresistance, and the acquisition of CSC properties in PDAC (Satoh et al., 2015; Wang et al., 2015; Zheng et al., 2015; Krebs et al., 2017; Aiello et al., 2018; Recouvreux et al., 2020).

**TABLE 1:** Overview of the individual TFs and their effects upon elevation in primary PDAC tumors. Influence on cellular identity, subtype, tumor characteristics and biological processes are highlighted. An upward pointing arrow (↑) indicates promoting effects, a downward pointing arrow (↓) inhibitory effects, a minus symbol (−) nor promoting nor inhibitory effects. Blank cells reflect that the process was not analyzed or no conclusion could be drawn from the indicated studies.

		Cellular identity		Subtype		Tumor characteristics		Biological processes					
TF	Context	Epithelial	Mesenchymal	Pancreatic progenitor	Squamous	Growth/Progression	Metastasis	Proliferation	Stemness	EMT	Invasion	Migration	References:
Yap1					↑	↑		↑					Kapoor et al. (2014), Lomberk et al. (2018)
GLI2		↓	↑	↓	↑				↑	↑			Adams et al. (2019)
ETV1		↓	↑			↑	↑	-		↑	↑		Heeg et al. (2016)
MAZ		↓	↑						↑	↑	↑	↑	Maity et al. (2018)
KLF7						↑	↑				↑	↑	Gupta et al. (2020)
SMAD4	SMAD4^+/+^	↓	↑			↓				↑			Bardeesy et al. (2006), Ischenko et al. (2021)
	SMAD4^−/−^	↑	↓	↑	↓	↑		-		↓			
RUNX3	SMAD4^+/−^					↑	↓	↑			↓	↑	Whittle et al. (2015)
	SMAD4^−/−^					↑	↑			↓			
TGIF1		↑	↓			↓	↓	-	↓	↓	↓	↓	Weng et al. (2019)
PDX1		↑	↓	↑	↓	↑	↓	↑		↓	↓		Collisson et al. (2011), Roy et al. (2016), Lomberk et al. (2018)
GATA6		↑	↓	↑	↓	↓	↓	↑		↓	↓		(Martinelli et al., 2016, 2017, Lomberk et al. (2018)
FOXA1		↑	↓	↑		↑	↑	↑		↓	↑	-/↓	Song et al. (2010), Diaferia et al. (2016), Martinelli et al. (2017), Roe et al. (2017)
FOXA2		↑	↓	↑				↑		↓		↓	Song et al. (2010), Bailey et al. (2016), Martinelli et al. (2017)
HNF4α		↑	↓	↑	↓	↓	-	↓					Bailey et al. (2016), Camolotto et al. (2021)
HNF1α								↓					Hoskins et al. (2014), Luo et al. (2015)
					↓	↑		↑	↑		↑	↑	Abel et al. (2018), Subramani et al. (2020)
SIX1				-	-	↑		↑					Camolotto et al. (2021)
SIX4				↓	↑	↑		↑					
BACH1		↓	↑			-	↑	-		↑	↑	↑	Sato et al. (2020)
ZEB1		↓	↑	↓	↑	-	↑	↑	↑	↑	↑		Krebs et al. (2017)
SNAI2		↓	↑		↑	↑	↑			↑	↑	↑	Recouvreux et al. (2020)
SOX2		↓	-					↑	↑	↑			Herreros-Villanueva et al. (2013)
PRRX1A		↑	↓	↑	↓		↑	↑		↓	↓		Takano et al. (2016)
PRRX1B		↑	↓	↓	↓		↑			↑	↑		

Apart from the activation of signaling cascades downstream of KRAS driving the subtype transition, the squamous subtype shows enrichment for TGFβ signaling (Bailey et al., 2016; Lomberk et al., 2018). In pre-malignant cells TGFβ acts as a tumor suppressor by inducing cell-cycle arrest, differentiation, and apoptosis (Massagué, 2008). Disruption of TGFβ signaling in PDAC prevents the tumor-suppressive effects, while activation of TGFβ signaling in a progressed state is a potent inducer of EMT (Dardare et al., 2020). A central player in canonical TGFβ signaling is SMAD4, whose inactivation or loss occurs as a late event during PDAC progression (Wilentz et al., 2000). The role of Smad4 in progression and metastasis remains controversial, as *Smad4*-deficiency attenuates EMT, leads to upregulated E-cadherin protein levels, and promotes a well-differentiated PDAC phenotype in the *Kras* ^*G12D*^;*Ink4a/Arf* ^*∆*^ mouse model (Bardeesy et al., 2006; Ahmed et al., 2017; Shichi et al., 2019). Moreover, simultaneous knockout of *Smad4* and *Kras* reduces EMT-related genes and promotes a Ras signaling signature (Ischenko et al., 2021). In contrast, other studies show that the loss of *SMAD4* is associated with shorter overall survival and a squamous phenotype (Blackford et al., 2009; Yamada et al., 2015). Interestingly, the TF Runx3 promotes metastatic colonization but is dependent on the *Smad4* state (Whittle et al., 2015). Heterozygous *Smad4* inactivation in the KPC mouse model promotes progression and growth of the primary tumor, while loss of the remaining wild-type allele leads to a highly metastatic disease. Furthermore, the expression of TGFβ target genes can be repressed by elevated TGIF1, potentially decreasing PDAC progression as demonstrated by HEK-293 cell transfection experiments and in PDAC mice (Seo et al., 2004; Weng et al., 2019). In summary, the intricate balance among several TFs involved in TGFβ signaling and their mutational status determine the impact on progression.

On the other hand, several endodermal lineage specifiers promote the pancreatic progenitor molecular subtype in PDAC, such as GATA6, FOXA1/A2, and HNF4α, identified *in silico* (Bailey et al., 2016; Roe et al., 2017; Brunton et al., 2020). Although *PDX1* expression is often increased during ADM and tumor onset, its downregulation or loss is mainly observed in poorly-differentiated tumors correlated with EMT and metastasis (Roy et al., 2016). Hence, high expression of *PDX1* is observed in the pancreatic progenitor subtype and well-differentiated tumors (Ischenko et al., 2014; Roy et al., 2016; Lomberk et al., 2018). Dysregulation of these TFs by inactivating mutations or repression can affect the EMT-equilibrium towards a more squamous identity. As an example, loss of *Gata6* in a mouse PDAC model decreases the *Cdh1* inducers Foxa1/a2 and de-represses EGFR signaling in favor of dedifferentiation (Martinelli et al., 2016, 2017). In contrast, FOXA1 elevation was identified in patients’ primary lesions and well-differentiated low-grade tumors, in part by activating *HNF4A* and other endodermal lineage specifiers (Duncan et al., 1998; Diaferia et al., 2016; Roe et al., 2017). Together with Gata6, Foxa1/a2 block EMT and promote epithelial differentiation in GEMM PDAC models (Song et al., 2010; Martinelli et al., 2017). Hence, direct transcriptional repression of *FOXA1* by BACH1 is required for metastatic colonization of AsPC-1 PaCa cells in an orthotopic implantation model (Sato et al., 2020). Interestingly, loss of *FOXA1/A2* is frequently detected in the squamous subtype and is sufficient to induce EMT in human PaCa cell lines (Song et al., 2010; Roe et al., 2017). Apart from repressing *FOXA1*, BACH1 activates *SNAI2,* which further promotes EMT, assessed by gene inactivation in human cell lines (Sato et al., 2020). Transcriptomic analysis on primary tumors and patient-derived cell lines revealed that during tumorigenesis HNF4α directly activates *HNF1A*, and loss of the former enables a transition towards a more squamous phenotype (Brunton et al., 2020; Camolotto et al., 2021). Moreover, HNF4α directly represses the mesodermal and neural differentiation TFs *SIX1/4*, whose elevated expression was linked to the squamous subtype (Camolotto et al., 2021). Downregulation of *HNF1A* is observed in the tumor vs. normal pancreas, suggesting that decreased HNF1α levels are important for PDAC tumor progression (Hoskins et al., 2014; Luo et al., 2015). Overexpression of *HNF1A* decreases cell-doubling times, while its knockdown significantly increases proliferation *in vitro*. HNF1α downregulates apoptosis inhibitors and modulates the expression of cell cycle genes. However, whether HNF1α acts through the AKT/mTOR pathway requires additional investigation, since silencing of *HNF1A* activates AKT/mTOR signaling, but may also result in reduced expression of PI3K, AKT and mTOR (Hoskins et al., 2014; Luo et al., 2015; Subramani et al., 2020). Other studies indicate that *HNF1A* is an oncogene necessary for the regulation of cancer stem cell (CSC) populations in PDAC, promotes anchorage-independent growth, proliferation, as well as invasive and migratory capacities (Abel et al., 2018; Subramani et al., 2020). These contradictory findings could be explained by the hypothesis that cellular plasticity and thus the ability to induce partial-EMT is indeed necessary to acquire stemness, whereas reversal to an epithelial phenotype is crucial for metastatic outgrowth at secondary sites. Conclusively, the expression of several TFs involved in specifying pancreatic cell-fate maintain the pancreatic progenitor subtype by (in)directly promoting epithelial-identity markers and inhibiting EMT/dedifferentiation (Table 1). Their downregulation abrogates these effects, allowing a switch towards the more aggressive squamous subtype (Figure 3).

#### 3.2.2 Induction of Epithelial-Mesenchymal Transition and Metastasis

Comparisons between primary PDAC tumors and matched metastasis revealed no specific metastasis-inducing genetic mutations, hinting towards gene regulatory mechanisms affecting late PDAC progression and metastasis (Campbell et al., 2010; Yachida et al., 2010; Makohon-Moore et al., 2017). The involvement of EMT-TFs in PDAC invasion and metastasis was initially questioned due to experimental challenges to observe EMT and the metastatic cascade *in vivo*. In particular, depletion of either Twist or Snail in the KPC mouse model of PDAC is not affecting metastasis formation, indicating that they are dispensable for this process (Zheng et al., 2015). However, depletion of Zeb1 in the same mouse model suppresses metastasis formation as well as experimental lung colonization capacity, stemness, and cell and metabolic plasticity (Krebs et al., 2017). Moreover, glutamine depletion promotes metastasis of orthotopically and intravenously injected KPC cells through induction of EMT by upregulation of *Snai2* via ERK signaling and ATF4 activation (Recouvreux et al., 2020). Collectively, these findings and research on core EMT-TFs in other cancers show that their individual contribution to invasion and metastasis is highly dependent on the cellular context (Elloul et al., 2005; Imamichi et al., 2007; Caramel et al., 2013; Denecker et al., 2014; Fischer et al., 2015; Zheng et al., 2015; Krebs et al., 2017; Stemmler et al., 2019; Recouvreux et al., 2020).

Liver metastases in KPC mouse models show elevated expression of Foxa1 and Prrx1a, whereas the expression of these factors decreases when the primary tumor acquires more squamous-associated features during progression (Takano et al., 2016; Roe et al., 2017). The decrease during progression and elevated expression in liver metastases suggests that re-expression of TFs associated with the pancreatic progenitor subtype is essential for successful liver colonization. Moreover, it was shown that Prrx1a enhances self-renewal, decreases invasiveness, and promotes metastatic outgrowth (Takano et al., 2016). Isoform b, on the other hand, fosters invasion, EMT, and dedifferentiation by promoting *Hgf* expression, suggesting that both isoforms distinctively regulate EMT and MET to form overt metastases. In addition, these two isoforms can form homo- and heterodimers, affecting transcriptional activity in human PaCa cell lines (Marchand et al., 2019). Simultaneous inactivation of *Snai1* and *Twist* induces a shift of the EMT-equilibrium to a more epithelial-like state in the primary tumor of KPC mice while enhancing liver metastases (Carstens et al., 2021). Sca1 and Pdx1 levels are also regulating metastatic capacities: Sca1^-^ cell lines derived from the KPC mouse model express elevated levels of Pdx1 and successfully metastasize in lungs and lymph nodes upon tail vein injections, whereas Sca1^+^ cells with lower levels of Pdx1 fail to metastasize (Ischenko et al., 2014). These findings support the idea that the reinforcement of epithelial features and thus cellular plasticity are required for metastatic competence (Figure 3).

### 3.3 Chromatin Dynamics and Epigenetic Regulation of Pancreatic Ductal Adenocarcinoma

In addition to the deregulation of established TF networks in tumor progression, epigenetic mechanisms of gene regulation are altered and become hijacked by cancer cells resulting in global changes in gene expression (Box 2). Genomic analyses in human PDAC revealed that up to 10% of mutations are identified in chromatin remodeling genes (Hayashi et al., 2021). Moreover, the epigenetic landscape of PDXs revealed that the squamous and pancreatic progenitor subtype can also be classified by patterns in DNA methylation and gene regulatory elements (Nicolle et al., 2017; Lomberk et al., 2018). Deregulation of specific histone modification enzymes in PDAC can lead to the transition towards the more aggressive squamous subtype by altering the chromatin states. Specifically, mutations in histone lysine demethylase 6a (*KDM6A*) combined with p53 alterations were associated with the squamous subtype of PDAC (Bailey et al., 2016). Loss of *KDM6A* alone is sufficient to induce a squamous-like subtype through activation of ΔNp63 (*TP63*), *MYC*, and *RUNX3* enhancer regions (Andricovich et al., 2018). Interestingly, upregulation of ΔNp63 alone is able to reprogram the enhancer landscape towards the squamous subtype by installing H3K27ac near genes promoting this subtype (Somerville et al., 2018). Upregulation of the histone methyltransferase *Nsd2* increases the global accumulation of the activation mark H3K36me2, thereby enriching the squamous gene signature in the KPC model. In contrast, loss of *Nsd2* decreases H3K36me2, resulting in enrichment of markers of the pancreatic progenitor subtype (Yuan et al., 2020). These findings suggest that the accumulation of dimethylation at H3K36 is necessary for cells to undergo EMT. Moreover, H3K36me2 may induce alterations in the enhancer landscape as its decrease leads to loss of H3K27ac in the same domains. Interestingly H3K36me2 transcriptionally affects the enhancer activity and thus the expression of most EMT-TF genes (*Zeb1/2*, *Snai1*, and *Twist2*) and of other metastasis-promoting TFs (Yuan et al., 2020). Histone methyltransferase EZH2 is part of the polycomb repressor complex 2 (PRC2) to set H3K27 methylation marks (Viré et al., 2006; Völkel et al., 2015). During pancreas regeneration, EZH2 transcriptionally represses *NFATC1,* whereas during tumorigenesis it induces *NFATC1* to drive KRAS-mediated PaCa plasticity (Chen et al., 2017). Making use of uncoupling Ezh2-NFATc1 regulation by combining conditional *Nfatc1* activation with *Ezh2* inactivation in *Kras* ^*G12D*^ mice, Patil et al. recently showed that partial loss of *Ezh2* leads to more differentiated PDAC tumors and fewer liver metastases in line with higher EZH2 protein expression in human high-grade tumors (Patil et al., 2021). Strikingly the most abundant negatively regulated target of Ezh2 is *Gata6*, a key regulator of endodermal identity. Moreover, re-expression of wild-type *Ezh2* abrogates *Gata6* expression in *Ezh2*-deficient cells. Conclusively, alterations in histone modification enzymes affect the chromatin states and aid in PDAC progression by inducing or repressing genes involved in PDAC progression.

BOX 2 **Epigenetic regulation of gene expression.**

Epigenetic mechanisms control the accessibility for TFs and the transcription machinery to selective regions of the genome. Consequently, depending on the state of the epigenetic landscape, TFs can bind to *cis*-regulatory elements to regulate gene transcription (Shen and Laird, 2013; Klemm et al., 2019). These mechanisms can be broadly divided into: post-translational histone modifications, DNA/RNA modifications (*e.g.* methylation) and non-coding RNAs (Shen and Laird, 2013; Lu et al., 2020). Histone modifications at specific regulatory regions include methylation and acetylation predominantly at histone H3 sites K4, K9, and K27 and are associated with active genes/promoters (H3K4me3), active/poised enhancers (H3K4me1), polycomb-repressed regions (H3K27me3) or heterochromatin (H3K9me3). These post-translational marks are set by a group of histone modifications enzymes, which are reviewed elsewhere (Bannister and Kouzarides, 2011). Super-enhancers (SEs) are a special type of enhancer which have been first identified in embryonic stem cells with clusters of TF binding sites for Sox2, Oct4 and Nanog (Hnisz et al., 2013; Whyte et al., 2013). SEs have also been identified in cancer and represent large regions of chromatin (up to 20 kb) that are densely clustered with enhancers, highly enriched for TF binding sites (Hnisz et al., 2013; Whyte et al., 2013). Their function is crucial in shaping cellular identity by regulating cell-type specific gene expression in both normal and diseased states (Hnisz et al., 2013).

Very recently, a strong contribution to PDAC progression was observed by the regulation of so-called super-enhancers (SEs) (Box 2) (Andricovich et al., 2018; Lomberk et al., 2018; Somerville et al., 2018). Comparisons between healthy cells and related cancer cells revealed that these SEs accumulate close to the loci of oncogenes during cancer progression, thus playing an important role in tumorigenesis (Hnisz et al., 2013). Profiling of the SE landscape revealed that several TFs transcriptionally regulate the expression of genes associated with the pancreatic progenitor subtype by binding upstream of these SEs (*i.e.* GATA6, FOS, FOXP1, FOXP4, KLF4, ELF3, NFIX, CUX1, and SSBP3) (Lomberk et al., 2018). These SEs mainly regulate genes of TFs associated with pancreas development (including *HNF1A*, *HNF4A,* and *PDX1*) and lipid metabolism. Epigenomic mapping of PDXs and PaCa cell lines showed that ΔNp63 activates squamous-specific SEs, including those near *FAT2*, *NECTIN1,* and *HIF1A* loci (Hamdan and Johnsen, 2018). Depletion of ΔNp63 reduces the H3K27ac at those SEs, indicating the dependency of ΔNp63 for writing these H3K27 acetylation marks. Loss of *KLF5* leads to a reduction in H3K27ac and H3K4me1 near these (super-)enhancers, inducing activation of stem cell- and mesenchymal-associated genes (Diaferia et al., 2016). *KLF5* was shown to be selectively expressed in well-differentiated human PDAC tumors and is required to maintain the expression of epithelial identity genes. Moreover, binding of KLF5 to enhancers increased the binding of ELF3 and FOXA1, which are both associated with low-grade PDAC tumors, demonstrating how KLF5 contributes to the regulation of pancreatic progenitor identity (Diaferia et al., 2016).

Progressive loss of repressive marks in large heterochromatin domains (H3K9 and H4K20) and increased H3K9, H3K27, and H4K16ac were found in distant metastases in comparison to the primary tumor, which helped to follow the acquisition of malignant traits (McDonald et al., 2017). These traits included resistance to oxidative stress, promoting a poorly-differentiated state, upregulation of DNA repair genes, and downregulation of oncogenic signal transduction in distant lung metastases. Comparisons of the epigenetic landscape from matched tumor and metastasis-derived organoids of the KPC model revealed that metastatic transition is accompanied by prominent changes in H3K27 acetylation, predominantly in enhancer regions. These changes are controlled by Foxa1, which is upregulated in metastases and was shown to cooperate with Gata5 for enhancer activation (Roe et al., 2017). Epigenetic regulation is also required to overcome the tumor-suppressive effects of TGFβ signaling, *i.e.*, the induced senescence and apoptosis before it can act as a trigger of EMT induction. Strikingly, NFATc1 elevation is crucial to overcome TGFβ-induced growth arrest by antagonizing H3K27ac and activation of TGFβ target genes including *Birc5*, *Ccnd1,* and *Plk1* (Hasselluhn et al., 2019) ***.***

Altogether, these findings indicate that epigenetic states define the molecular subtypes of PDAC in a highly dynamic process. Alterations in the epigenetic landscape including SEs are key features in PDAC progression towards malignancy, supporting the acquisition of cellular plasticity.

## 4 Novel Therapeutic Approaches to Target Transcription Factors

Despite the advances in therapies, non-metastatic local PDAC eligible for surgical resection followed by adjuvant chemotherapy remains the sole curative option, applicable for only 10–20% of patients (Gillen et al., 2010; Werner et al., 2013; Benassai et al., 2015; Orth et al., 2019). First-line treatment options for patients with locally advanced or distant metastatic PDAC are usually limited to conventional chemotherapies. Despite changes in treatment regimens from monotherapies to multi-agent chemotherapies, the survival rates of PaCa patients remain largely unchanged and success is severely limited due to *de novo* acquisition or pre-existing resistance. Various intrinsic and extrinsic tumor feature alterations have been proposed contributing to drug resistance, including the microenvironment, altered metabolism, EMT, and the presence of CSCs (Grasso et al., 2017; Swayden et al., 2018; Tuerhong et al., 2021). The lack of blood vessels and the abundant desmoplasia create a hypoxic and nutrient-scarce environment, forcing PDAC cells to alter their metabolism to sustain proliferation (Sousa and Kimmelman, 2014; Yang et al., 2020). In addition, these microenvironmental features impede therapeutic delivery (Neesse et al., 2011; Dufort et al., 2016). In general, global efforts are made to design precision therapies to combat PDAC, including therapeutic targets to inhibit tumor-intrinsic pathways such as KRAS, PI3K, AKT, mTOR, JAK/STAT, SHH, NOTCH, and WNT signaling cascades (Chandana et al., 2019). These strategies predominantly target mediators in oncogenic signaling cascades upstream of TFs, thereby indirectly affecting the expression of deregulated TFs. So far, the only precision medicine approved for PDAC treatment is erlotinib, a potent inhibitor of EGFR-related kinase, used in combination with gemcitabine (Moore et al., 2007; Sinn et al., 2017).

As summarized before, PDAC is highly plastic, and inhibition of certain kinases can be compensated by the dysregulation of other effectors (as seen for KRAS) and often converge to the same set of TFs (*e.g.* ZEB1/2, Snail, Slug, Twist, and SOX2) (Figure 3). Hence, these TFs are attractive therapeutic targets. For example, silencing of *ZEB1* restores the expression of epithelial markers and resensitizes PaCa cells to standard chemotherapy (Arumugam et al., 2009; Wellner et al., 2009; Meidhof et al., 2015). Similarly, treatment with the HDAC inhibitor Mocetinistat *in vitro* and upon xenotransplantation upregulates ZEB1-repressed target genes, particularly miR-200 and miR-203, reducing ZEB1 protein expression and restoring drug sensitivity (Meidhof et al., 2015). Moreover, knockout of *Snai1* or *Twist* increased the sensitivity to erlotinib and gemcitabine (Zheng et al., 2015). Although initially thought to be undruggable, recent attempts to design and identify drugs that target TFs are promising (Henley and Koehler, 2021). Strategies to inhibit TFs directly and indirectly include targeting the expression level, modulating proteasomal degradation, disrupting protein/protein interactions, and ligand/DNA binding abilities (Lambert et al., 2018; Bushweller, 2019).

Multiple clinical trials are currently ongoing to evaluate therapeutics that indirectly target TF expression levels in PDAC. Studies on triptolide (TPL), a diterpenoid triepoxide, shows promising results in PDAC cell lines and orthotopic pancreatic cancer models (Borja-Cacho et al., 2010; Chugh et al., 2012; Zhao et al., 2020). TPL binds to XPB, a subunit of TFIIH, thereby inhibiting transcription globally (Vispé et al., 2009; Titov et al., 2011). Moreover, treatment with TPL (Minnelide) leads to a rapid downregulation of *MYC* gene expression and protein levels (Titov et al., 2011). Phase II clinical trials are currently ongoing to study the effect of TPL on non-responsive PDAC tumors. In addition, clinical attempts to target epigenetic deregulation are currently under evaluation. These include treatment with Azitidine and/or Romideposin, in combination with immuno- as well as standard chemotherapeutic treatments in patients with surgically resected and advanced PDAC. The inhibition of effectors upstream of TFs may pose severe problems in non-neoplastic cells, as these pathways are often indispensable for proper cell functioning. It is hypothesized that direct TF-targeting approaches minimize the side effects by precisely modulating their deregulated transcriptional programs (Henley and Koehler, 2021). The drug COTI-2, a thiosemicarbazone, has been shown to directly convert mutant p53 to the wild-type 3D structure. *TP53* is approximately mutated in 72% of all PDAC (Raphael et al., 2017). Gain-of-function mutations in *TP53* increase the aggressiveness in PaCa and promotes metastasis (Morton et al., 2010; Weissmueller et al., 2014). Additionally, it negatively regulates the PI3K/AKT/mTOR pathway (Salim et al., 2016; Robertson et al., 2020). Phase I clinical trials are currently ongoing to study the effect of COTI-2 as monotherapy or with combinations for the treatment of malignancies. Moreover, bi-weekly treatment of pretreated metastatic PDAC patients with the STAT3 inhibitor BBI608, shows promising activity (Bekaii-Saab et al., 2016).

The advent of targeted therapies to target TF together with the advances in other therapeutic strategies (*e.g.* immunotherapy, targeting receptors, membrane transporters, and enzymes) and rise of precision medicine bear the promise to improve PDAC patient outcomes. Although the field is still evolving, these combinational treatments offer valuable options for PaCa patients to overcome acquired therapy resistance and aggressive phenotypes.

## Glossary

∆NP63: deltaNp63 alternate variant of the tumor protein p63
3D: three dimensional
ACTA2 (αSMA): actin alpha 2, smooth muscle
ADM: acinar-ductal metaplasia
AKT/PKB: AKT serine/threonine kinase 1/protein kinase B
ALDH1: aldehyde dehydrogenase 1
ATF4: activating transcription factor 4
BACH1: BTB domain and CNC homolog 1
BIRC5: baculoviral IAP repeat containing 5
BMP: bone morphogenetic protein
BRCA2: breast cancer type 2 susceptibility protein
CCND1: cyclin D1
CDH1: cadherin 1/(epithelial) E-cadherin
CDH2: cadherin 2
CDKN2A: cyclin dependent kinase inhibitor 2A
CDX2: caudal type homeobox 2
CK14: cytokeratin 14
CK19: cytokeratin 19
CRAF/Raf1: Raf-1 proto-oncogene, serine/threonine kinase
CSC: cancer stem cell
CUX1: cut like homeobox 1
DNA: deoxyribonucleic acid
E2F7: E2F transcription factor 7
EGF: epidermal growth factor
EGFR: epidermal growth factor receptor
ELF3: E74 like ETS transcription factor 3
EMT: epithelial-mesenchymal transition
EPCAM: epithelial cell adhesion molecule
ERK: extracellular signal-regulated kinases
ESRP1: epithelial splicing regulatory protein 1
ETV1: ETS variant transcription factor 1
EZH2: enhancer of zeste 2 polycomb repressive complex 2 subunit
FAT2: FAT atypical cadherin 2
FBP1: fructose-bisphosphatase 1
FGF2: fibroblast growth factor 2
FN: fibronectin 1
FOS: Fos proto-oncogene, AP-1 transcription factor subunit
FOXA1: forkhead box protein A1
FOXA2: forkhead box protein A2
FOXP1: forkhead box P1
FOXP4: forkhead box P4
GATA4: GATA binding protein 4
GATA6: GATA binding protein 6
GCG: glucagon
GEMM: genetically engineered mouse model
GLI2: GLI family zinc finger 2
GLIS3: GLIS family zinc finger 3
HAS2: hyaluronic acid synthase 2
HDAC: histone deacetylase
HES1: hairy and enhancer of split 1
HGF: hepatocyte growth factor
HIF1A: hypoxia inducible factor 1 subunit alpha
HKDC1: hexokinase domain containing 1
HNF1α: hepatocyte nuclear factor 1 alpha
HNF1β: hepatocyte nuclear factor 1 beta
HNF4α: hepatocyte nuclear factor 4 alpha
HNF6 (Onecut1): hepatocyte nuclear factor 6 (one cut family member 1)
IFN: interferon
INK4A/ARF: cyclin-dependent kinase 4 Inhibitor/alternative reading frame
INS1: insulin I
INS2: insulin II
INSM1: insulinoma-associated 1/INSM transcriptional repressor 1
IPMN: intraductal papillary mucinous neoplasia
ISL1: islet 1/ISL LIM homeobox 1
JAK: janus kinase
KDM6A: lysine demethylase 6A
KITLG: KIT ligand
KLF: krüppel-like factor
KRAS: KRAS proto-oncogene, GTPase
MAFA: musculoaponeurotic fibrosarcoma oncogene homolog A/MAF bZIP transcription factor A
MAFB: musculoaponeurotic fibrosarcoma oncogene homolog A/MAF bZIP transcription factor B
MAPK: mitogen-activated protein kinase
MAZ: MYC associated zinc finger protein
MCN: mucinous cystic neoplasm
MET: mesenchymal-epithelial transition
miRNA: microRNA
MIST1: muscle, intestine and stomach expression 1/basic helix-loop-helix family member A15 (BHLHA15)
MMP: matrix metalloprotease
MNX1: motor neuron and pancreas homeobox 1
MPC: multipotent progenitor cell
mTOR: mammalian target of rapamycin
MYC: MYC proto-oncogene, bHLH transcription factor
NECTIN1: nectin cell adhesion molecule 1
NEUROD1: neuronal differentiation 1
NFATC1: nuclear factor of activated T cells 1
NFATC4: nuclear factor of activated T cells 4
NFIX: nuclear factor I X
NF-κB: nuclear factor kappa B
NGN3: neurogenin 3
NKX2.2: NK2 homeobox 2
NKX6.1: NK6 homeobox 1
NR5A2: nuclear receptor subfamily 5 group A member 2
NSD2: nuclear receptor binding SET domain protein 2
p21(CIP/WAF1): cyclin-dependent kinase inhibitor 1/wild-type p53-activated fragment 1
p53/TP53: tumor protein p53
p63/TP63: tumor protein p63
PaCa: pancreatic cancer
PAK: p21 activated kinase
PanIN: pancreatic intraepithelial neoplasia
PAX4: paired box 4
PAX6: paired box 6
PcG: polycomb group
PDAC: pancreatic ductal adenocarcinoma
PDGF: platelet-derived growth factor
PDX: patient-derived tumor xenograft
PDX1: pancreatic and duodenal homeobox 1
PI3K: phosphoinositide 3-kinases
PLK1: polo like kinase 1
POU3F4: POU class 3 homeobox 4
PRC2: polycomb repressor complex 2
PROX1: prospero homeobox 1
PRRX1: paired-related homeodomain transcription factor 1
PTF1A/p48: pancreas associated transcription factor 1a
RASA3: RAS p21 protein activator 3
RBPJL: recombination signal binding protein for immunoglobulin kappa J region like
RFX3: regulatory factor X3
RFX6: regulatory factor X6
RNA: ribonucleic acid
RUNX3: RUNX family transcription factor 3
SCA1: stem cell antigen 1
SE: super-enhancer
SHH: sonic hedgehog
SIX1: SIX homeobox 1
SIX4: SIX homeobox 4
SLUG/SNAI2: snail family transcriptional repressor 2
SMAD4/DPC4: SMAD family member 4/deleted in pancreatic cancer 4
SNAIL/SNAI1: snail family transcriptional repressor 1
SOX2: SRY-box transcription factor 2
SOX9: SRY-box transcription factor 9
SPARC: secreted protein acidic and cysteine rich
SPP1: secreted phosphoprotein 1
SSBP3: single stranded DNA binding protein 3
STAT3: signal transducer and activator of transcription 3
TF: transcription factor
TFIIH: transcription factor IIH
TGFα: transforming growth factor alpha
TGFβ: transforming growth factor beta
TGIF1: TG-interacting factor 1
TJP1/ZO-1: tight junctional protein 1/zonula occludens 1
TPL: triptolide
TWIST1: twist-related protein 1
VIM: vimentin
WNT: portmanteau from wingless and int-1 (locus of frequent mouse mammary tumor virus (MMTV) integration)
XPB/ERCC3: xeroderma pigmentosum type B/ERCC excision repair 3, TFIIH core complex helicase subunit
YAP: Yes1 associated transcriptional regulator
ZEB1: zinc finger E-box binding homeobox 1
ZEB2: zinc finger E-box binding homeobox 2
